# *Capsosiphon fulvescens* glycoprotein inhibits AGS gastric cancer cell proliferation by downregulating Wnt-1 signaling

**DOI:** 10.3892/ijo.2013.2079

**Published:** 2013-08-23

**Authors:** YOUNG-MIN KIM, IN-HYE KIM, TAEK-JEONG NAM

**Affiliations:** Institute of Fisheries Sciences, Pukyong National University, Ilgwang-myeon, Gijang-gun, Busan 619-911, Republic of Korea

**Keywords:** *Capsosiphon fulvescens*, Wnt-1, β-catenin, cell cycle

## Abstract

Previously, we examined various apoptosis pathways in the AGS gastric cancer cell line using *Capsosiphon fulvescens* glycoprotein (Cf-GP). In this study, we focused on the downregulation of the Wnt-1 signaling pathway and cell cycle arrest. Upregulation of the Wnt signaling pathway has been observed in various cancer cells. The Wnt signal ligand acts in both canonical and non-canonical pathways. Among them, Wnt-1 was dependent on the canonical pathway. Here, we show inhibition of Wnt-1 signaling, β-catenin and transcription factors in AGS cells via Cf-GP. First, we examined the Frizzled receptor and Wnt-1 signal-related proteins including Axin, LRP, β-catenin, APC and GSK-3β. In addition, the expression levels of transcription factors Tcf/LEF were determined by western blot analysis and RT-PCR. Based on the data, we confirmed downregulation of the Wnt-1 signaling pathway by Cf-GP. Also, we determined the expression levels of cell cycle-related proteins cyclin D and c-myc, and looked for cell cycle arrest by cell cycle test analysis. We found that AGS cells arrested in the G0/G1 phase by Cf-GP. These results provide a mechanism of AGS cell inhibition through the downregulation of Wnt-1 signaling by Cf-GP.

## Introduction

The Wnt ligand binds immediately to its receptor gene, Frizzled, and this binding site is divided into the canonical and non-canonical pathways (1,2). Among them, the canonical pathway is dependent on β-catenin and affects cancer cell adhesion. Wnt-1 proteins are secreted from cells. Thereafter, Wnt-1 protein combines with Frizzled proteins and lipoprotein-related protein 5/6 (LRP 5/6) of the two receptor molecules. The Wnt-1 signaling pathway is dependent on β-catenin (3–6). Wnt proteins bind the cysteine-rich glycoprotein, acting only in a limited range of ligands, and focal activation of the receptor-mediated signal transduction system acts as an important regulator of cell proliferation and differentiation factors (7). β-catenin is a factor related to cell adhesion. In normal cells, downregulation of Wnt-1 signaling degrades β-catenin in the Axin-APC protein complex by GSK-3β. However, cancer cells show upregulation of Wnt-1 signaling. Therefore, cancer cells continue to grow and metastasis occurs. The β-catenin degradation complex, an Axin degradation complex, inhibits the activity of GSK3β when Wnt stimulation is external. Therefore, phosphorylation of β-catenin is inhibited (8). Additionally, normal cells have operating E-cadherin in the nucleus, but cancer cells degrade E-cadherin. E-cadherin in the nucleus inhibits β-catenin and is involved in intercellular adhesion, which plays an important role in the maintenance of normal epithelial cells and epithelial structures (9); catenin in the cytoplasm and formation of the cadherin/catenin complex is involved, and intercellular adhesion fails to progress to invasive carcinoma (10,11). Change in cancer cell grade increases metastasis when the expression of E-cadherin is lost (12). Additionally, Snail, typically a zinc finger transcription factor, inhibits E-cadherin expression, which inhibits the cytosolic level of β-catenin in the nucleus. Similar to Snail genes that are resistant to apoptosis, invasion and migration of cancer cells promote the epithelial-mesenchymal transition (EMT) and induce increased Snail levels in the nucleus through upregulation of Wnt-1 signaling (13). Therefore, β-catenin is not degraded and cytoplasmic β-catenin levels increased, which stabilize β-catenin by moving to the nucleus, and the Tcf/LEF transcription factor regulates the expression of various genes. Increases in cancer cell proliferation through the upregulation of cell cycle-related proteins, c-myc and cyclin D, occur via activated Tcf/LEF (14–17). In addition, representative β-catenin target genes increase cell proliferation of ICAM-1 and c-jun by increasing the expression of β-catenin and Tcf/LEF (18,19). In a previous study, we confirmed apoptosis of AGS cells by *Capsosiphon fulvescens* (20). Also, Cf-GP induced downregulation of TGF-β1 signaling pathway in AGS cells. Therefore, we observed inhibition of AGS cell proliferation through MTS assay (21). In this study, we observed the downregulation of Wnt-1 signaling by Cf-GP and inhibition of AGS cell proliferation by a G0/G1 phase arrest.

## Materials and methods

### Preparation of Cf-GP

The *C. fulvescens* (Cf) used in this experiment was purchased in 2010 in Korea. Forty grams of Cf powder was diluted with 1 liter of water. The Cf powder in diluted water was stirred for 3 h at 80°C in heating mantle and centrifuged at 1,500 × g for 15 min at 4°C. Next, three volumes of 95% ethanol were added, and precipitation was removed by vacuum filtration. Supernatants were added to 80% ammonium sulfate and stirred for 24 h. Salt composition was then removed through a dialysis membrane (Por Membrane MW 3,500 Da, Spcectrum Laboratories Inc., Rancho Dominguez, CA, USA) for 1 day at 4°C. The concentrated solution was then distributed into a 1.5-ml tube and stored at −70°C until use. These samples were named *C. fulvescens* glycoprotein (Cf-GP).

### Cell culture

AGS human gastric cancer cell line (American Type Culture Collection, Manassas, VA, USA) was maintained at 37°C in a 5% CO_2_ humidified atmosphere. Cells were cultured in RPMI-1640 medium with 10% fetal bovine serum (FBS; Hyclone, Logan, UT, USA), 100 U/ml penicillin and 100 mg/ml streptomycin. Cells were cultured to 80% confluence in 100-mm diameter dishes. The RPMI-1640 medium was replaced every day.

### Cell attachment analysis

The cell attachment analysis was performed using 3 ml per 100-mm diameter dish in sterile 1% gelatin at 120°C for 20 min and then coated after storage at 4°C. Cells were cultured in RPMI-1640 medium with 10% FBS and grown to 80% confluence in 100-mm diameter dishes coated with 1% gelatin. Next, cells were treated with Cf-GP of various concentrations and cultured for 24 h. The unattached (apoptotic) cells were stained with trypan blue and live cells were confirmed by microscopy at ×200 magnification.

### mRNA expression analysis

AGS cells were seeded onto 6-well plates at 2×10^4^ cells/well in 2 ml of medium. Cells were maintained at 24 h and the medium was replaced with SFM. After 24 h, the SFM was replaced with Cf-GP (5, 10 or 20 *μ*g/ml) for 24 h. Cells were treated with 1 ml TRIzol reagent (Invitrogen, Carlsbad, CA, USA), and cDNA was synthesized using the Oligo(dT) primer (iNtRON Biotechnology Inc., Seongnam, Korea). The synthesized cDNA was added to 2X TOPsimple™ DyeMIX-nTaq (Enzynomics Inc., Daejeon, Korea), and the primer (Table I) was added to 0.1% diethylpyrocarbonate (DEPC) water. PCR products were separated on a 1% agarose gel and stained with RedSafe nucleic acid staining solution (iNtRON Biotechnology Inc).

### Western blot analysis

AGS cells were cultured in 100-mm diameter dishes. Cells were grown to 80% confluency and the medium was then replaced with SFM for 4 h. The medium was then replaced with fresh SFM containing Cf-GP (5, 10 or 20 *μ*g/ml) for 24 h. For collection, cells were washed with phosphate-buffered saline (PBS) and then added to extraction lysis buffer (20 mM Tris, pH 7.5, 150 mM NaCl, 1 mM EDTA, 1 mM EGTA, 1% Triton X-100, 2.5 mM sodium pyrophosphate, 1 mM β-glycerophosphate, 1 mM sodium orthovanadate, 1 *μ*g/ml aprotinin, 1 *μ*g/ml leupeptin, 1 *μ*g/ml pepstatin A, 0.25% Na-deoxycholate and 1 mM PMSF). To examine protein expression, western blot analysis was performed by separating proteins on a 10–15% sodium dodecyl sulfate polyacrylamide gel electrophoresis (SDS-PAGE), and proteins were then transferred onto an Immobilon-P transfer membrane (Millipore Co., Billerica, MA, USA). The transferred membrane was blocked at room temperature with 1% bovine serum albumin in TBS-T (10 mM Tris-HCl, pH 7.5, 150 mM NaCl and 0.1% Tween-20), and then shaking with the indicated primary antibodies (diluted 1:1,000): anti-Wnt-1, anti-Frizzled, anti-LRP, anti-APC, anti-Axin, anti-GSK-3β, anti-β-catenin, anti-E-cadherin, anti-Snail, anti-LEF-1, anti-Tcf, anti-ICAM-1, anti-c-jun, anti-c-myc or anti-cyclin D from Santa Cruz Biotechnology (Santa Cruz Biotechnology Inc., Santa Cruz, CA, USA). The secondary antibodies, horseradish peroxidase-conjugated goat, mouse and rabbit antibody (1:10,000) from GE Healthcare Bio-Sciences (Piscataway, NJ, USA) were then added. The reaction was terminated using SuperSignal West Pico Chemiluminescent substrate (Thermo Fisher Scientific Inc., Rockford, IL, USA) and visualized by X-ray film (Kodak, Rochester, NY, USA).

### Cell cycle analysis

The rate of cell cycle phase by Cf-GP treatment was determined using a Muse™ cell cycle kit from Millipore (EMD Millipore Co., Billerica, MA, USA). Cells were cultured onto 6-well plates grown to 80% confluency and treated with SFM or various concentrations of Cf-GP (5, 10 or 20 *μ*g/ml). After 24 h of treatment, cells were collected in 1% FBS-RPMI-1640 media and Muse cell cycle test reagent was added to each tube. Cells were mixed by vortexing and the reaction was performed for 30 min at room temperature in the dark. Following treatment, cells were stained to determine G0/G1, S and G2/M phase rates of the cell cycle using a Muse cell analyzer (EMD Millipore Co., Hayward, CA, USA).

### Statistical analysis

Results of this experiment were compared using SPSS ver.10.0 Programs (SPSS Inc., Chicago, IL, USA), and validated with analysis of variance (ANOVA) and Duncan’s multiple range tests. A p-value <0.05 was considered to indicate a statistically significant difference. All data are expressed as the mean ± standard deviation (SD).

## Results

### Effect of Cf-GP on the cell attachment assay and apoptosis staining

**Figure 1.**
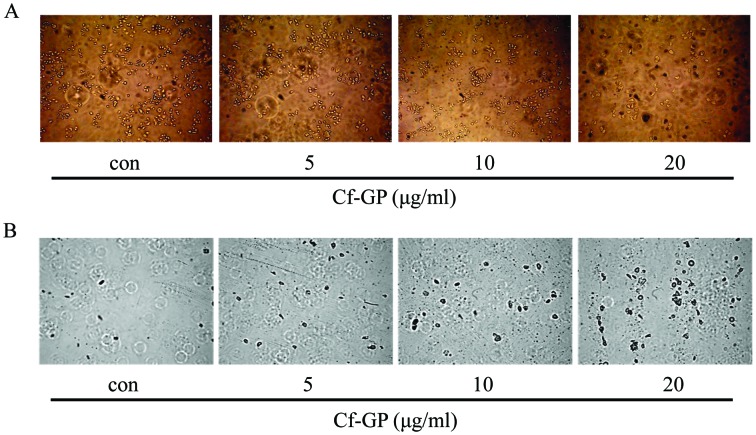
Attachment assay and trypan blue staining. (A) Attachment of AGS cells was determined by 1% gelatin-coated plates. Cells were cultured in 10% FBS and grown to 80% confluence in 100-mm diameter dishes coated with 1% gelatin. Cells were then treated with Cf-GP (5, 10 or 20 *μ*g/ml) for 24 h. (B) Unattached apoptotic cells were stained with trypan blue, and live cells were confirmed by microscopy (×200 magnification).

Live cell motility and cell attachment analysis were performed by coating 1% gelatin on 100-mm diameter dishes. In addition, apoptotic cells were stained with trypan blue. Gelatin was used as a representative extracellular matrix (ECM) component, and motility of active cancer cells were well attached to the gelatin (22). The cells were seeded onto 1% gelatin-coated 100-mm diameter dishes and treated with Cf-GP for 24 h. As a result, we observed decreased attachment of AGS cells in a dose-dependent manner upon treatment with Cf-GP (Fig. 1A). In general, apoptotic cells are permeable. Therefore, dead cells will stain with trypan blue and viable cells will not (23). As shown in Fig. 1B, the group treated with Cf-GP displayed increased apoptotic cells compared to the control group.

### Effect of Cf-GP on the downregulation of Wnt-1, Frizzled and LRP

**Figure 2.**
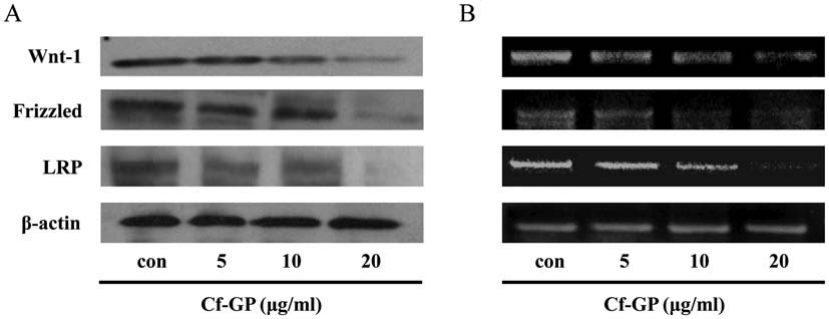
Effects of Cf-GP on the expression levels of Wnt-1, Frizzled and LRP5. Cells were treated with Cf-GP (5, 10 or 20 *μ*g/ml) for 24 h. Genes expression was determined by western blot analysis and RT-PCR. (A) Western blot analysis using anti-Wnt-1, anti-Frizzled, anti-LRP and anti-β-actin antibodies. SDS-PAGE was performed on acrylamide gel. (B) cDNA and primers were synthesized. PCR was then performed at the indicated annealing temperatures. Reaction products were electrophoresed on a 1% agarose gel and visualized with RedSafe reagent.

The Wnt-1/Frizzled protein receptor demonstrates that cell proliferation is associated with cancer cells (24). Therefore, inhibition of cancer cell proliferation and attachment is required to suppress Wnt-1/Frizzled/LRP. We observed the expression levels of these genes by western blot analysis and RT-PCR. Cells were treated with various concentration of Cf-GP (5, 10 or 20 *μ*g/ml) for 24 h. As a result, we observed a decrease in protein levels of Wnt-1, Frizzled and LRP in a dose-dependent manner upon Cf-GP treatment (Fig. 2A). Additionally, mRNA expression levels revealed the same results (Fig. 2B). Expression of a sub-factor of Wnt-1 signaling was due to reduced expression of a receptor that was also observed in these experiments.

### Effect of Cf-GP on the downregulation of Wnt-1 signaling

**Figure 3.**
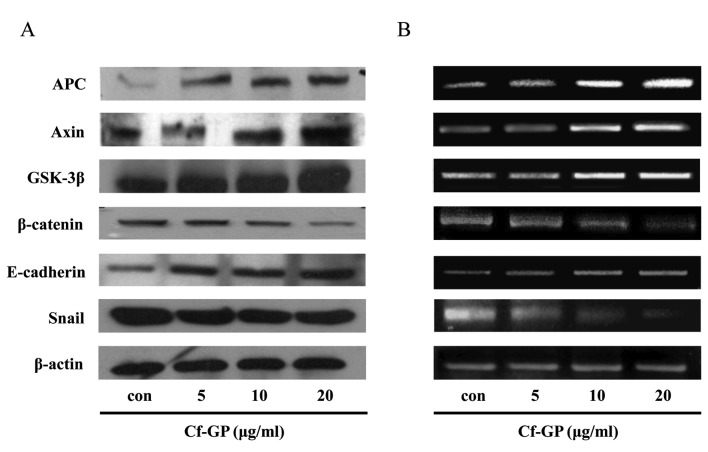
Effects of Cf-GP on the expression levels of APC, Axin, GSK-3β, E-cadherin and β-catenin. Cells were treated with Cf-GP (5, 10 or 20 *μ*g/ml) for 24 h. Gene expression was determined by western blot analysis and RT-PCR. (A) Western blot analysis using anti-APC, anti-Axin, anti-GSK-3β, anti-E-cadherin, anti-β-catenin, anti-Snail and anti-β-actin antibodies. SDS-PAGE was performed on acrylamide gels. (B) cDNA and primers were synthesized. PCR was then performed at the indicated annealing temperatures. Reaction products were electrophoresed on a 1% agarose gel and visualized with RedSafe reagent.

The Wnt-1 receptor was activated in cancer cells and altered the various sub-factors. Previously, we confirmed inhibition of Wnt-1 and Frizzled and LRP receptors. Therefore, expression levels of the sub-factors of these genes (APC, Axin, GSK-3β and β-catenin) were measured in the same manner. The β-catenin was degraded by APC, Axin and the GSK-3β complex in normal cells. In addition, APC and Axin induced inhibition of Wnt-1. However, these genes did not operate in cancer cells by stimulated Wnt-1 and mutant Axin, and LRP combined with Wnt-1 activation. As a result, we confirmed increased expression of APC and Axin. In addition, GSK-3β increased to decompose the role of β-catenin by Cf-GP. Thus, a reduction in β-catenin is involved in cell adhesion. Additionally, we observed increased E-cadherin, which is involved in β-catenin inhibition and decreased Snail. E-cadherin induced degration of β-catenin in normal cells. However, almost all cancer cells have low level of E-cadherin by upregulation of Snail. So, β-catenin degradation does not occur in cancer cells (Fig. 3).

### Effect of Cf-GP on the inhibition of transcription factors and cell cycle genes

**Figure 4.**
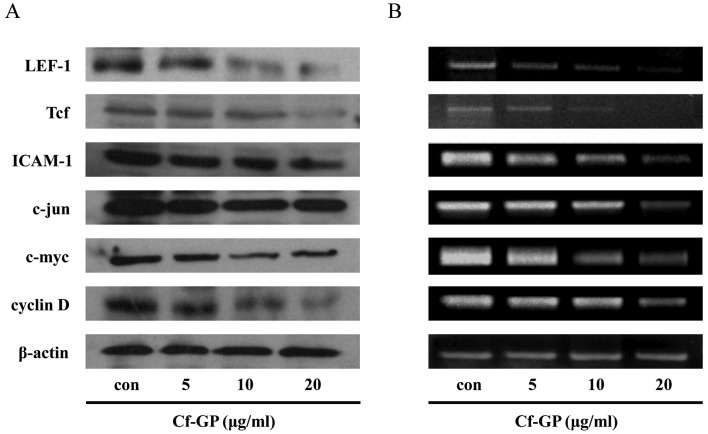
Effects of Cf-GP on the expression levels of Tcf, LEF-1, ICAM-1, c-jun, c-myc and cyclin D. Cells were treated with Cf-GP (5, 10 or 20 *μ*g/ml) for 24 h. Gene expression was determined by western blot analysis and RT-PCR. (A) Western blot analysis using anti-Tcf, anti-LEF-1, anti-ICAM-1, anti-c-jun, anti-c-myc, anti-cyclin D and anti-β-actin antibodies. SDS-PAGE was performed on acrylamide gel. (B) cDNA and primers were synthesized. PCR was then performed at the indicated annealing temperatures. Reaction products were electrophoresed on a 1% agarose gel and visualized with RedSafe reagent.

Target gene expression occurs following accumulation of β-catenin in the nucleus and entering of the Tcf/LEF binding factor. This promotes transcription of c-myc and cyclin D, which promotes cell division by the β-catenin/Tcf/LEF complex. Like many cancer cells, the expression of these genes does not arrest the cell cycle. Therefore, we examined the expression levels of LEF, Tcf, ICAM-1, c-jun, c-myc, and cyclin D by western blot analysis and RT-PCR. Cells were treated with Cf-GP (5, 10 or 20 *μ*g/ml) for 24 h. Next, cells were cultured under the same conditions as the previous experiments. As a result, we observed the downregulation of protein and mRNA levels of these genes in a dose-dependent manner with Cf-GP (Fig. 4).

### Effect of Cf-GP on G0/G1 arrest of the AGS cell cycle

**Figure 5.**
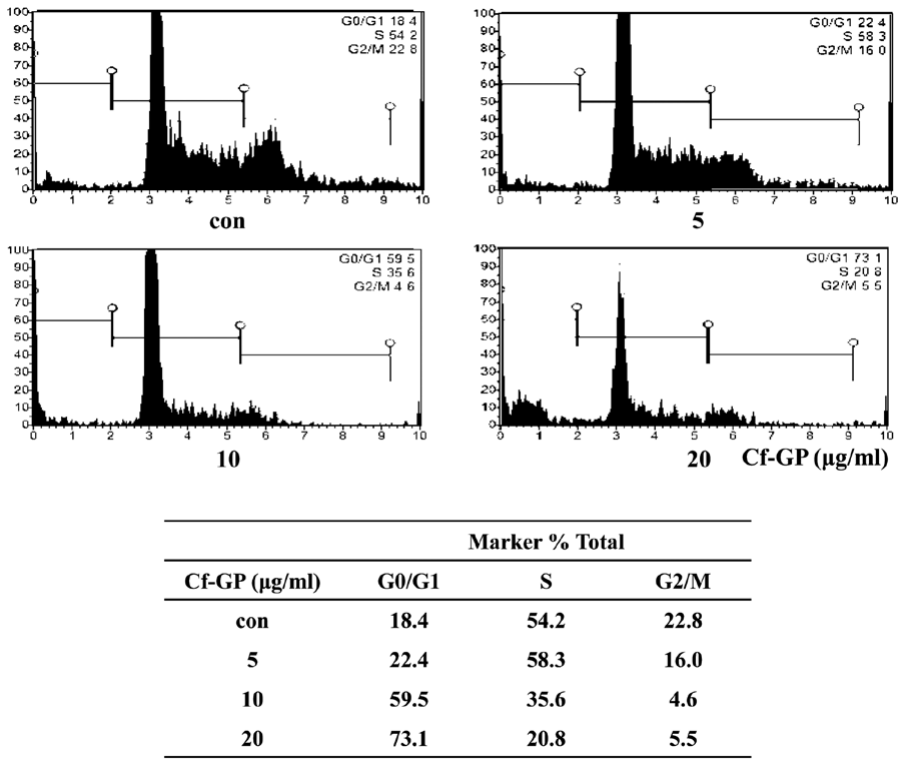
Effect of Cf-GP on cell cycle progression in AGS cells. Cells were treated with SFM (control groups) or Cf-GP (5, 10 or 20 *μ*g/ml) for 24 h. DNA was stained with propidium iodide, and flow cytometric analysis of cell phase distribution was performed using a Muse cell analyzer.

Expression of c-myc and cyclin D did not properly adjust Wnt-1 signal transduction in cancer cells. Therefore, the cancer was generated by the promotion of proliferation and division of cells. The order and progress of cell cycle events were monitored during checkpoints of the cell cycle that occur during the transition from G0/G1 to S phase. Progression of the cell through the four phases (G0/G1-S-G2/M) of the cell cycle is regulated by sequential activation. We used a cell cycle test following the Muse cell cycle kit protocol and treated cells with Cf-GP (5, 10 or 20 *μ*g/ml) for 24 h. As a result, arrest of G0/G1 phase with final concentration group (20 *μ*g/ml) was increased approximately 50% compared to the control group in AGS cells (Fig. 5).

## Discussion

Wnt proteins are involved in the cysteine-rich secreted ligand coupled receptor-mediated signal transduction pathway. The Wnt pathway is activated by binding to the cell membrane receptor through autocrine or paracrine signals. The original Wnt signal controls the development of organs, cell proliferation, morphology and motility within vertebrates. The Wnt pathway can be divided into two types. The protein family has a composition of cysteine-rich glycoproteins that play important role in proliferation and differentiation of cells. However, Wnt stimulation causes the growth and proliferation of various cancer cells (25,26). The Wnt signaling pathway has separated into at least 19 types of Wnt genes involved in the canonical and non-canonical pathways. One Wnt pathway is dependent on β-catenin, also known as the ‘canonical Wnt pathway’, which is activated by Wnt-1, 2, 3a and 10a, and decides the fate of cells. The alternate β-catenin-independent pathway, is activated by Wnt-4, 5a and 11, which is referred to as the ‘non-canonical Wnt pathway’ or ‘Wnt/calcium pathway’; this pathway is involved in cell polarity, adhesion and shape (27,28). In this study, we focused on cell inhibition, cell motility and proliferation through decreased β-catenin by downregulation of Wnt-1. Normally, secreted Frizzled-related proteins (SFrp) bind Wnt-1 proteins to induce and interfere with Wnt, Frizzled, and the LRP complex in cells. Therefore, Wnt signaling will not occur through the degradation of β-catenin by Axin, APC and GSK-3β complex. However, cancer cells cannot operate the Axin/APC/GSK-3β complex by overexpression of Wnt-1. So, β-catenin was not degraded and thus accumulated in the nucleus, thereby promoting expression of the transcription factor. In a previous study, we observed inhibition of AGS cell proliferation through MTS assay by Cf-GP (5, 10 or 20 *μ*g/ml) (21). Decreased cell proliferation was evident upon Cf-GP treatment (5, 10 or 20 *μ*g/ml) in a dose-dependent manner, and treatment at high concentration of 20 *μ*g/ml led to an approximately 40% reduction compared to the untreated group. In this study, apoptotic events were confirmed by staining cells with trypan blue. Trypan blue moves into the cell by diffusion and exocytosis through adenosine triphosphate (ATP) from viable cells that are discharged to the outside of the cells and back. Living cells do not allow trypan blue to enter cells. Therefore, apoptotic cells stained with trypan blue by diffusion and motility was degraded. As shown in Fig. 1, inhibition of motility and induction of apoptosis was observed by Cf-GP (5, 10 or 20 *μ*g/ml) in a dose-dependent manner.

Next, we confirmed the expression levels of Wnt-1-related genes by western blot analysis and RT-PCR. As a result, Wnt-1 and receptor proteins (Frizzled and LRP) decreased in a dose-dependent manner upon treatment with Cf-GP (Fig. 2). In addition, based on the above results, various gene factors were measured through downregulation of Wnt-1 by Cf-GP. The expression levels of β-catenin decreased based on western blot analysis and RT-PCR. Additionally, E-cadherin and the Axin/APC/GSK-3β complex levels increased in a dose-dependent manner by Cf-GP. This explains that upregulation of E-cadherin and the Axin/APC/GSK-3β complex inhibits nucleus accumulation of β-catenin. Also, we observed increased E-cadherin by downregulation of Snail (Fig. 3). In the absence of β-catenin degradation, its accumulation in the nucleus induced activation of transcription factors (Tcf/LEF), which can have a significant effect on cell proliferation (29,30). Therefore, it is important to inhibit transcription factors (Tcf/LEF) in cancer cells. In a previous experiment, we observed the inhibition of β-catenin and Wnt-1. Therefore, we expected a reduction by expression of the Tcf/LEF transcription factor activated in cancer cells, as well as an increase in the expression of a variety of factors including cyclin D, c-myc, ICAM-1 and c-jun, which are involved in the promotion of cancer cell cycle and proliferation (31,32). In the case of cell cycle-related genes, Cf-GP inhibits cyclin D, c-myc, ICAM-1 and c-jun in AGS cells (Fig. 4). We then confirmed a change in cell cycle phase, which was monitored during cell culture with Cf-GP for 24 h. As shown in Fig. 5, the G0/G1 phase was approximately 18.4% in the control groups, but Cf-GP treatment groups induced an increase in G0/G1 arrest (22.4, 59.5 and 73.1%, respectively). Therefore, the AGS cell cycle was stagnant, and apoptosis was induced by Cf-GP in a dose-dependent manner. *C. fulvescens,* green algae that grows in Korea and other Asian coastal countries, has long been a healthy food and bioactive material. Our previous results showed an anticancer effect by Cf-GP through Fas signaling and apoptosis of AGS cancer cells (33). In this study, we confirmed the inhibition of AGS gastric cancer cell migration by downregulating the Wnt-1 signaling pathway via Cf-GP. Therefore, our results suggest potential for functional food and therapeutic use of Cf-GP.

## Figures and Tables

**Table I.** Oligonucleotide sequences of primer pairs used for RT-PCR.

Name	Sequence of primers (5′→3′)
Wnt-1	S: TGC-ACG-CAC-ACG-CGC-GTA-CTG-CAC
	A: CAG-GAT-GGC-AAG-AGG-GTT-CAT-G
Frizzled	S: CAG-AGC-GGG-GCA-GCA-GTA-CAA
	A: GCG-CGG-GCA-GGA-GAA-CTT
LRP5	S:CAG-CAC-CCG-GAA-GAT-CAT-TGT
	A:TCG-TTG-ATC-TCG-GTG-TTG-ACC
APC	S: TAT-CTT-CAG-AAT-CAG-CCA-GGC-AC
	A: AAA-GTA-TCA-GCA-TCT-GGA-AGA-ACC
Axin	S: ACC-GAA-AGT-ACA-TTC-TTG-ATA-AC
	A: TCC-ATA-CCT-GAA-CTC-TCT-GC
GSK-3β	S: CAG-CAA-GGT-GAC-AAC-AGT-GG
	A: GGA-ACA-TAG-TCC-AGC-ACC-AGA
β-catenin	S: GAA-ACG-GCT-TTC-AGT-TGA-GC
	A: CTG-GCC-ATA-TCC-ACC-AGA-GT
E-cadherin	S: GAA-CAG-CAC-GTA-CAC-AGC-CCT
	A: GCA-GAA-GTG-TCC-CTG-TTC-CAG
Snail	S: TAT-GCT-GCC-TTC-CCA-GGC-TTG
	A: ATG-TGC-ATC-TTG-AGG-GCA-CCC
Tcf	S: TGA-CCT-CTC-TGG-CTT-CTA-CT
	A: TTG-ATG-GTT-GGC-TTC-TTG-GC
LEF-1	S: CCA-GCT-ATT-GTA-ACA-CCT-CA
	A: TTC-AGA-TGT-AGG-CAG-CTG-TC
ICAM-1	S: CAC-CTC-CTG-TGA-CCA-GCC-CA
	A: AAC-AGG-ACG-GTC-GCT-GAG-GG
c-jun	S: ATG-ACT-GCA-AAG-ATG-GAA-ACG
	A: TCA-AAA-TGT-TTG-CAA-CTG-CTG-CG
c-myc	S: CCA-GGA-CTG-TAT-GTG-GAG-CG
	A: CCT-GAG-GAC-CAG-TGG-GCT-GT
Cyclin D	S: CTG-GCC-ATG-AAC-TAC-CTG-GA
	A: GTC-ACA-CTT-GAT-CAC-TCT-CC
β-actin	S: CGT-ACC-ACT-GGC-ATC-GTG
	A: GTG-TTG-GCG-TAC-AGG-TCT-TTG

S, sense; A, antisense.
